# Acetaldehyde Adsorption Characteristics of Ag/ACF Composite Prepared by Liquid Phase Plasma Method

**DOI:** 10.3390/nano11092344

**Published:** 2021-09-09

**Authors:** Byung-Joo Kim, Kay-Hyeok An, Wang-Geun Shim, Young-Kwon Park, Jaegu Park, Heon Lee, Sang-Chul Jung

**Affiliations:** 1 Department of Carbon & Nanomaterials Engineering, Jeonju University, Jeonju 55069, Korea; kimbj2015@gmail.com (B.-J.K.); khandragon@jj.ac.kr (K.-H.A.); 2 Department of Chemical Engineering, Sunchon National University, Suncheon 57922, Korea; wgshim@sunchon.ac.kr; 3 School of Environmental Engineering, University of Seoul, Seoul 02504, Korea; catalica@uos.ac.kr; 4 Department of Environmental Engineering, Sunchon National University, Suncheon 57922, Korea; worn0623@gmail.com (J.P.); honylee@hanmail.net (H.L.)

**Keywords:** liquid phase plasma, Ag/ACF composite, acetaldehyde, specific surface area, adsorption capacity, dose response model

## Abstract

Ag particles were precipitated on an activated carbon fiber (ACF) surface using a liquid phase plasma (LPP) method to prepare a Ag/ACF composite. The efficiency was examined by applying it as an adsorbent in the acetaldehyde adsorption experiment. Field-emission scanning electron microscopy and energy-dispersive X-ray spectrometry confirmed that Ag particles were distributed uniformly on an ACF surface. X-ray diffraction and X-ray photoelectron spectroscopy confirmed that metallic silver (Ag^0^) and silver oxide (Ag_2_O) precipitated simultaneously on the ACF surface. Although the precipitated Ag particles blocked the pores of the ACF, the specific surface area of the Ag/ACF composite material decreased, but the adsorption capacity of acetaldehyde was improved. The AA adsorption of ACF and Ag/ACF composites performed in this study was suitable for the Dose–Response model.

## 1. Introduction

Volatile organic compounds (VOC) is a generic term for liquid or gaseous organic compounds that can evaporate easily into the atmosphere because of their low boiling point. These compounds are diverse, ranging from solvents commonly used in industry to organic gases discharged from chemical and pharmaceutical factories or plastic drying processes [1,2]. Liquid fuels with low boiling points, paraffin, olefins, aromatic compounds, and other hydrocarbons are commonly used. Among them, acetaldehyde (AA) is one of 35 specific air-hazardous substances in Korea and has a reference concentration for trace emission of 0.01 ppm because it is harmful to the human body [3,4]. Acetaldehyde can cause respiratory paralysis, convulsion, and corneal injury [5].

Adsorption, photocatalytic oxidation, plasma oxidation, and the like are known as treatment methods for the removal of acetaldehyde. Since it has problems such as limited decomposition reaction, generation of by-products (photocatalytic oxidation), and generation of nitrogen dioxide and ozone (plasma oxidation), an adsorption method using an absorbent is generally used the most [6].

Carbon materials such as activated carbon (AC) have many advantages, such as high surface area, thermal stability, inexpensiveness, and renewability, and are therefore used as major adsorbents [5,7]. In addition, various metals have been supported on carbon materials and used to improve adsorption properties and effects [8]. Silver (Ag) is used widely because it improves the adsorption efficiency and has a sterilizing effect [9]. It is known that metal silver (Ag^0^) and silver ion (Ag^+^) supported on carbon materials form catalytically, oxidize for some organic compounds, or increase the oxidation potential to improve adsorption capacity [7,10].

The sol-gel method has been mainly used as a method of placing supporting metals on carbon materials. On the other hand, the liquid phase plasma (LPP) method has attracted recent attention [11,12]. The LPP process is a kind of cold plasma process in which plasma is generated by applying a high voltage to an electrode installed in a liquid [13,14]. Wastewater treatment, hydrogen production, nanoparticle synthesis, and the fabrication of various composites can be prepared using various radicals and electrons in the plasma field formed in the reaction aqueous solution [15,16]. The LPP method is easy to operate and can be prepared in a single step. In addition, it is an eco-friendly process because it does not generate secondary waste [17,18].

In this study, activated carbon fiber (ACF) was prepared using high-density polyethylene fiber as a raw material. Ag/ACF composite was synthesized by precipitating Ag nanoparticles in the prepared ACF using the LPP method. The acetaldehyde adsorption experiment was performed using Ag/ACF composite as an adsorbent, and the pure ACF and adsorption performance were compared and evaluated.

## 2. Materials and Methods

### 2.1. Materials

In this study, HDPE fiber was prepared by melt spinning of high-density polyethylene (HDPE, 2700 J, Melt index 7.0 g/10 min, Density 0.949 g/cm^3^, Lotte chemical Co., Seoul, Korea). ACF prepared by sulfur crosslinking, carbonization, and steam activation of HDPE fiber was used as a support. Sulfuric acid (98%, 7683-4100, DaeJung Chemical & Metal Co., Ltd., Gyeonggi-Do, Korea) was used as a crosslinking agent of HDPE fiber. Silver nitrate (AgNO_3_, Sigma-Aldrich, St. Louis, MO, USA) was used as a precursor of silver particles precipitated in ACF. Deionized water (DaeJung Chemical & Metal Co., Ltd., Gyeonggi-Do, Korea) with an electrical conductivity of 2 siemens or less was used to prepare the LPP aqueous reaction solution. All chemicals were used as received.

### 2.2. Preparation of Aactivated Ccarbon Fiber

**Figure 1:**
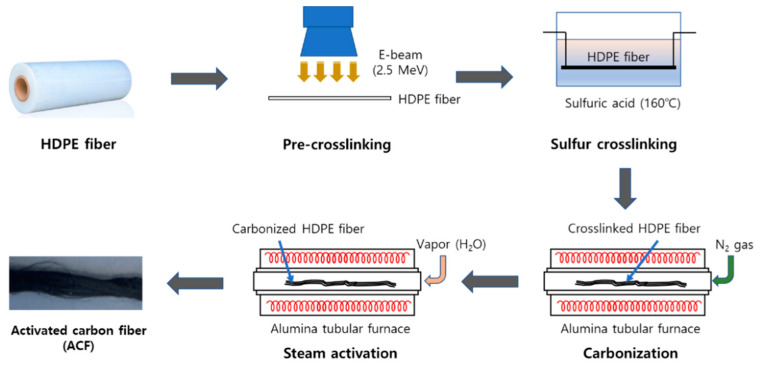
Preparing process diagram of activated carbon fiber using high-density polyethylene fiber.

Figure 1 shows the process of preparing ACF using HDPE fiber. The HDPE fiber was subjected to pre-crosslinking treatment through electron beam irradiation (EBI); the irradiated electron energy was 2.5 MeV, and the irradiation dose of the E-beam was 1000 kGy. A crosslink structure was formed by dipping 8 g of EBI-treated HDPE fiber in 600 mL of sulfuric acid at 160 °C for 60 min. Crosslinked HDPE fibers were washed with distilled water and dried in a vacuum oven at 70 °C for 24 h. The dried HDPE fiber was carbonized at 900 °C using a self-made alumina tubular furnace (1000 mm long and 100 mm in diameter). Carbonization was carried out for 60 min using nitrogen gas (10 mL/min) being supplied. Thereafter, the inlet gas was changed to water vapor (0.5 mL/min) to perform steam activation for 1 h, and after the activation reaction was completed, nitrogen gas (300 mL/min) was purged and cooled.

### 2.3. Preparation of Silver Nanoparticle Precipitated ACF

**Figure 2:**
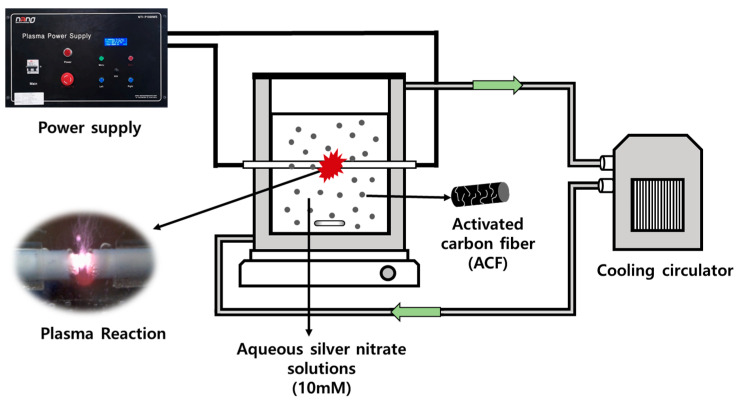
Schematic diagram of LPP method for preparation of Ag particle-precipitated ACF.

Figure 2 shows the LPP device (Nano Technology Co., Ltd., Daejeon, Korea) used in this study to prepare silver nanoparticle precipitated ACF (Ag/ACF composite). Electrical power was supplied to the tungsten electrodes installed in the center of the quartz reactor by using the power supply on the upper left. At this time, the pulse width was 5 μs, the applied voltage was 250 V, and the frequency was operated under operating conditions of 30 kHz. The LPP reactor was manufactured in a double tube type, and in order to prevent the temperature increase due to heat generated by plasma generation, cooling water was circulated in the outer channel of the reactor to keep the temperature of the reactant solution constant at 15 °C.

The Ag/ACF composite preparation process using the LPP method was performed in the following order [14,18]. The detailed configuration and specifications of the LPP system used in this experiment are described elsewhere. Since ACF prepared by the method shown in Figure 1 is difficult to disperse in the LPP reactant aqueous solution, it was pulverized to a small size using ball milling and dispersed in the solution. After ball milling ACF composite to a powder for 5 min, 0.5 g was mixed with 250 mL of deionized water. AgNO_3_, a Ag precursor, was added to the ACF-dispersed reactant aqueous solution at a certain concentration (5~15 mM) and stirred until completely dissolved. After transferring the prepared reactant solution to the LPP reactor, the LPP reaction was performed by supplying power to the tungsten electrodes fixed in the center of the reactor using a power supply. After the LPP reaction, the solid was centrifuged, followed by washing and filtration to obtain the Ag/ACF composite. The collected Ag/ACF composite was vacuum dried at 80 °C for 24 h to remove moisture. The number written on the back of the prepared Ag/ACF composite means the concentration of AgNO_3_ added to the reactant aqueous solution. For example, Ag/ACF-15 is Ag/ACF prepared by adding AgNO_3_ at a concentration of 15 mM.

### 2.4. Acetaldehyde Adsorption Experiment

For the acetaldehyde adsorption experiment, a special preparation was used in which acetaldehyde was added to nitrogen gas, a dilution gas, to make a concentration of 20 ppm. A self-contained fix-bed adsorption system was used for the acetaldehyde adsorption experiment, and the adsorbent (ACF and Ag/ACF composite) was placed in the center of a quartz tube (700 mm length, 25.4 mm diameter). The amount of adsorbent used was added constantly at 0.3 g. The flow rate and reaction temperature of the acetaldehyde reaction gas were kept constant at 400 mL/min and 20 °C, respectively. After collecting a 100 mL sample using a gas sampling pump (GV-100, GASTEC Corp., Kanagawa, Japan) of the reaction gas that has passed through the adsorption tube filled with adsorbent, the detector tube (No. 92L, GASTEC Corp., Kanagawa, Japan) was used to measure the acetaldehyde concentration.

### 2.5. Characterization of Ag/ACF Composite

The morphology and chemical composition of the Ag/ACF composite prepared using the LPP method were measured by field-emission scanning electron microscopy FE-SEM (JSM-7100F, JEOL Ltd., Tokyo, Japan) and energy-dispersive X-ray spectrometry (EDS). X-ray diffraction (X’Port PRO, PANalytical, Almelo, The Netherlands) was conducted to examine the crystallinity. The physicochemical properties of the Ag/ACF composite were measured by X-photoelectron spectroscopy (Multilab 2000 system, Thermo Fisher Scientific, Waltham, MA, USA) with non-monochromatic Al Ka radiation (1486.6 eV), and the N_2_-isotherm curve, surface area, and pore size distribution of ACF and Ag/ACF composite were measured using BELSORP-Max (BEL Japan, Tokyo, Japan).

## 3. Results

**Figure 3:**
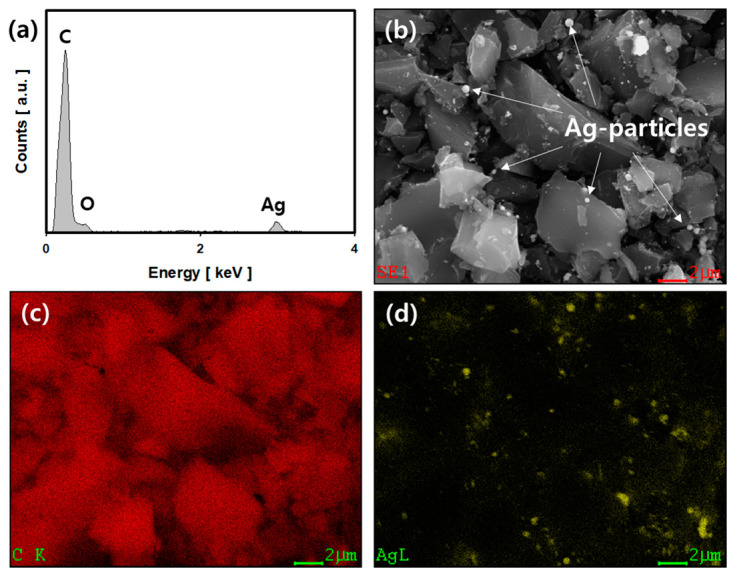
Analysis results of FE-SEM and EDS of Ag/ACF-15 composite prepared by the LPP method: (**a**) EDS spectrum, (**b**) FE-SEM real image and (**c**) carbon element mapping image, and (**d**) Ag element mapping image.

Figure 3 shows FE-SEM images, and the results of EDX analysis of Ag/ACF-15 composite prepared using the LPP method. Figure 3a shows the spectrum by EDS attached to the FE-SEM. The peaks at 0.25 keV and 0.53 keV were assigned to carbon (C Kα) and oxygen (O Kα). The peak at 2.98 keV was due to silver (Ag Lα). The composition of the Ag/ACF composite was 93.63 At.%, 4.83 At.%, and 1.54 At.% of carbon, oxygen, and silver, respectively. Figure 3b presents an FE-SEM real image of the Ag/ACF-15 composite. Ag particles were observed on the surface of ACF with a size of 200 nm to 500 nm. In the plasma field generated in the aqueous reaction solution, H_2_O_2_ is produced by active species, such as OH radicals. The Ag ions dissociated from the precursor and present in the aqueous reaction solution were reduced through the following reaction, converted to Ag particles, and precipitated on the ACF surface [19].

2Ag^+^ + H_2_O_2_ → 2 Ag↓+ 2H^+^ + O_2_ (1)

Figure 3c,d show the mapping images of C and Ag on the Ag/ACF-15 composite surface. In the case of Figure 3c, the carbon element exists in the same way as the ACF form, and the silver element in Figure 3d was detected at the same location as the Ag particles in Figure 3b.

Table 1 presents the atomic compositions of ACF and Ag/ACF composites calculated from the results of EDS analysis attached to FE-SEM. ACF consisted of the carbon element (C) and oxygen element (O) at 96.57 At.% and 3.43 At.% ratios, respectively. Ag/ACF compositions prepared using the LPP method showed that the Ag element increased in proportion to the injected Ag precursor concentration and, accordingly, the carbon element decreased. On the other hand, as the content of the Ag element increased, the At.% of oxygen also showed a tendency to increase.

**Figure 4:**
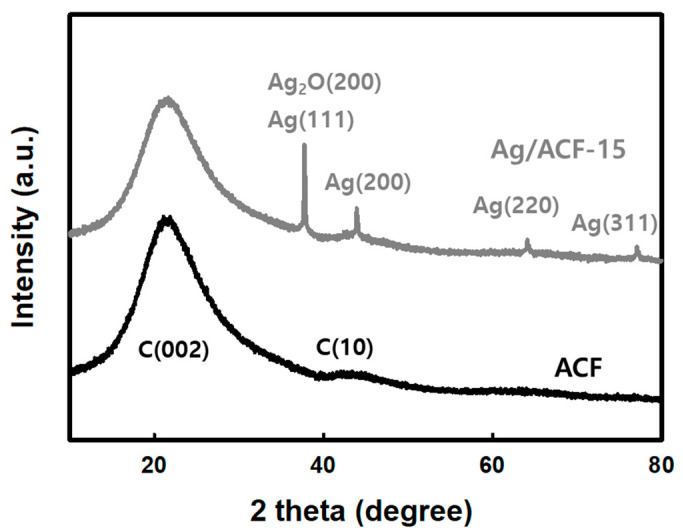
X-ray diffraction patterns of ACF and Ag/ACF-15 composite.

Figure 4 shows the XRD pattern of ACF and the Ag/ACF-15 composite. In the ACF in Figure 4, diffraction lines were observed at 21.3° and 43.5° at 2θ, which were assigned to the C (002) and (01) planes, respectively. With graphite, the diffraction line of the C (002) plane is generally observed at 25° 2θ, but the ACF used in this study shifted to a low 2θ. This means that the ACF used in this experiment has a wide space between the carbon lattice [20]. In the case of the Ag/ACF-15 composite shown above, four diffraction lines were also observed along with the C (002) and (10) planes shown in ACF. The strong peak at 38.1° 2θ is the diffraction line due to the metallic Ag (111) plane and the Ag_2_O (200) plane. The diffraction lines at 44.2°, 64.2°, and 77.4° 2θ were assigned to the Ag (200), Ag (220), and Ag (311) planes [21]. The Ag particles precipitated by the LPP method were a mixture of metallic silver (Ag^0^) and silver oxide (Ag_2_O), which was consistent with the XRD pattern of Figure 4.

**Figure 5:**
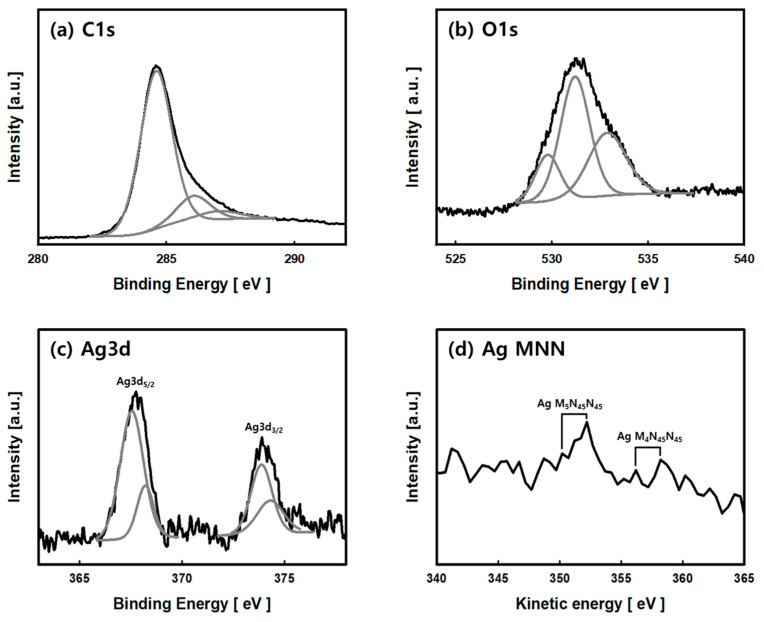
XPS spectra of the C1s region (**a**), O1s region (**b**), Ag3d region (**c**), and Ag NMM (**d**) of Ag/ACF-15 composite prepared by the LPP method.

Figure 5 shows the C1s, O1s, Ag3d, and Ag MNN XPS spectrum of the Ag/ACF-15 composite. In Figure 5a, C1s photoelectron line was fitted to three contributions. The peak at binding energy (BE) 284.6 eV was due to the graphite carbon C-C bond, the peak at BE 286.2 eV was due to the C-O bond, and the peak at BE 287.1 eV was due to the carbon of the C=O bond [22,23]. In the O1s region of Figure 5b, three fitted photoelectron lines were observed, and the weak binding energy peak of 529.8 eV was due to a Ag-O bond. The results in Figure 4 confirmed that the Ag precipitated on the ACF surface was in the form of metallic Ag and Ag_2_O crystals. Therefore, the peak is due to the Ag-O bond of Ag_2_O [24]. The peaks at 531.2 eV and 532.2 eV were assigned to oxygen bonded to the carbon of ACF-15 composite, such as C=O and C-OH or C-O-C bonds [25]. Figure 5c is the XPS result for the Ag3d region, and two doublet-fitted photoelectron lines were observed. The peaks observed at BE 367.6 eV and 373.6 eV were generated by Ag_2_O with an oxidation state of +1, and the peaks generated at BE 368.3 eV and 374.3 eV were peaks by metallic silver (Ag^0^) [26,27,28]. In addition, the spin orbital splitting between Ag_3_d_5/2_ and Ag_3_d_3/2_ of Ag_2_O and metallic silver was maintained at 6.0 eV [28]. Figure 5d is a Ag MNN spectrum, and a weak intensity peak was observed. Kinetic energy (KE) peaks at 350.2 eV and 352.2 eV are peaks by Ag M_5_N_45_N_45_ of Ag^+1^ and Ag^0^. Auger parameter (AP) was calculated using BE of Ag3d_5/2_ and KE of Ag MNN (Ag M_5_N_45_N_45_ and Ag M_4_N_45_N_45_), and AP of Metallic Ag was 720.5 eV (Ag3d_5/2_-Ag M_5_N_45_N_45_) and 726.4 eV (Ag3d_5/2_-Ag M_4_N_45_N_45_). The AP of Ag^+1^ was calculated to be 717.8 eV (Ag 3d_5/2_-Ag M_5_N_45_N_45_) and 723.7 eV (Ag3d_5/2_-Ag M_4_N_45_N_45_), and it was confirmed that it was due to silver ion of Ag_2_O [28]. Overall, XPS showed that the Ag particles precipitated by LPP were a mixture of metallic silver and silver oxide, which is consistent with the XRD pattern in Figure 4.

**Figure 6:**
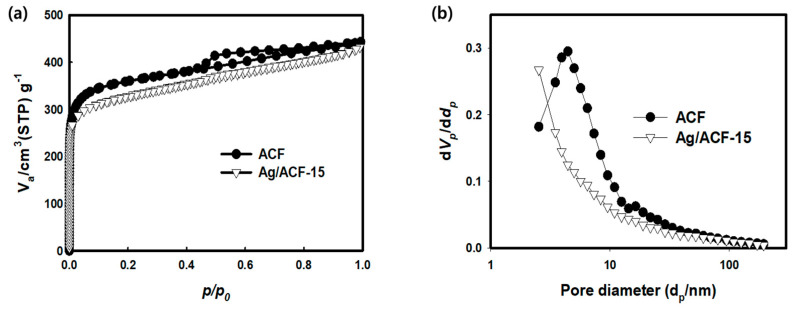
Adsorption–desorption isotherm curves (**a**) and pore size distribution (**b**) of ACF and Ag/ACF-15 composite.

Figure 6a shows the nitrogen adsorption–desorption isotherm curves of ACF and the Ag/ACF-15 composite measured under 77 K/N_2_ conditions. Both ACF and the Ag/ACF-15 composite showed a mixed form of a type-I and type-IV isotherm profile. In the case of ACF, a small H4 hysteresis loop was observed in the 0.5~0.8 P/P_0_ range. The Ag/ACF-15 composite curve showed a somewhat lower N_2_ adsorption behavior than the ACF curve, and the hysteresis loop of the mesopore section that appeared in ACF was reduced significantly. Ag particles precipitated by the LPP method affected the surface area. The surface area of Ag/ACF-15 composite measured by the Brunauer–Emmett–Teller (BET) method was 1264 m^2^/g, which was lower than that of the ACF (1462 m^2^/g). Ag particles precipitated by the LPP reaction had a blocking effect on the micro and mesopore structure of ACF, which reduced the surface area [29]. Figure 6b is the pore size distribution (PSD) of ACF and the Ag/ACF-15 composite measured using the Barrett–Joyner–Halenda (BJH) method. ACF mainly had a mesopore structure with a size of 2 to 50 nm. In the case of Ag/ACF-15, in which Ag particles were precipitated on the surface of the ACF by the LPP method, it was shown that the pores corresponding to the size of 2~30 nm were reduced.

Table 2 shows pore characteristics such as specific surface area, total pore volume, micropore volume, mesoporous volume, mesopore ratio, and pore diameter obtained through the BET and BJH method. The surface area, pore volume and mesopore ratio of Ag/ACF composites showed lower values than those of ACF, indicating that they were inversely proportional to the amount of Ag precipitated on the ACF surface. It is presumed that this is because the blocking effect of ACF on the pore inlet increases due to the increase in the Ag content precipitated through the LPP process. The average pore diameter of Ag/ACF composite was overall lower than that of ACF.

**Figure 7:**
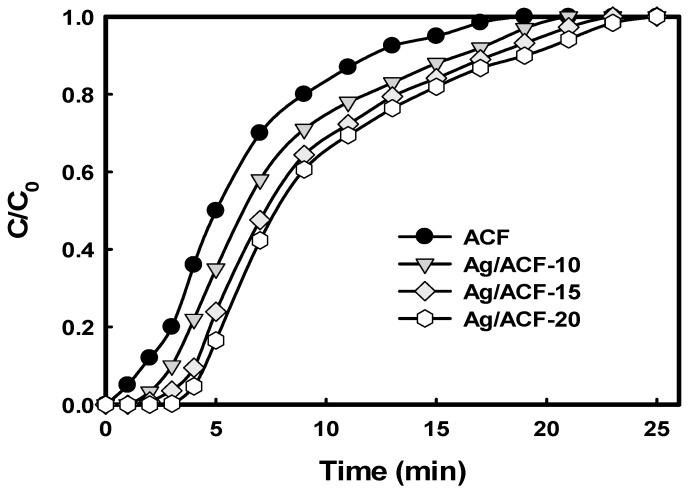
Acetaldehyde adsorption breakthrough curves of ACF and Ag/ACF composites.

Figure 7 shows the results of changes in AA concentration at the outlet of the adsorption reactor for each reaction time obtained from an AA adsorption experiment using ACF and Ag/ACF composites as an adsorbent. In both ACF and Ag/ACF composites, the concentration of AA at the outlet of the adsorption reactor increased slowly at the beginning of the reaction, but showed a tendency to increase rapidly after breakthrough. In addition, Ag-ACF composites showed higher AA adsorption performance compared to ACF. Ag precipitated on the surface of ACF decreased the surface area and pore volume, but improved the adsorption properties of acetaldehyde. It can be seen that these results are consistent with Table 2.

**Figure 8:**
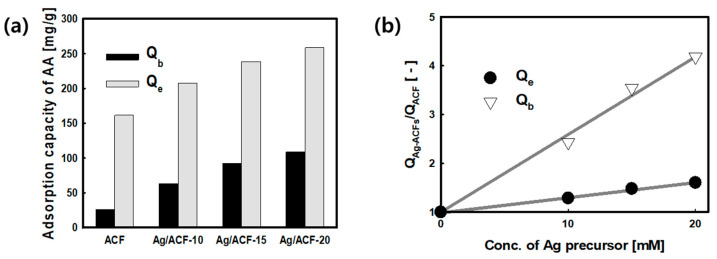
Breakthrough adsorption capacity (Q_b_) and exhaustion adsorption capacity (Q_e_) obtained from AA adsorption breakthrough curve (**a**) and relative adsorption capacity versus concentration of silver precursor (**b**).

Figure 8 and Table 3 show the AA adsorption capacity and column efficiency parameters obtained using the results of Figure 5. The gas flow rate Q at which AA was diluted was 400 mL/min, and the initial concentration of AA (C_0_) was 20 mg/L. The absorbent mass (*m*) was 0.3 g, and the height of absorbent (*Z*) was 5.0 mm. The breakthrough time (t_b_, C/C_0_ = 0.05) and exhaustion time (t_e_, C/C_0_ = 1.0) of Ag/ACF composites were obtained from 2.4 to 4.1 min and 21.0 to 25.0 min. Additionally, it can be seen that these values are higher than the t_b_ (1.0 min) and t_e_ (19.0 min) of ACF. At the breakthrough time and exhaustion time of ACF, the adsorption amount of AA was calculated to be 26.0 mg/g and 161.6 mg/g, respectively. The AA adsorption amounts of Ag/ACF composites prepared by the LPP process was 63.1~108.6 mg/g in t_b_ and 207.5~258.7 mg/g in t_e_. Ag/ACF composites increased 2.4~4.2 (t_b_) times and 1.3~1.6 (t_e_) times compared to ACF, indicating that the adsorption performance of AA by Ag particles was improved at breakthrough and exhaustion time. In addition, the height of the mass transfer zone was also reduced by 17 to 31% in Ag/ACF composites compared to ACF, suggesting that the fixed bed column due to low mass transfer resistance can be used more efficiently [30]. It was found that Ag particles precipitated on the surface of the ACF by the LPP reaction improved the AA adsorption ability, and the amount of Ag particles acted as a major influence factor on the adsorption performance improvement. This is presumed to be due to the high adsorption affinity between the Ag particles on the ACF surface and the carbonyl group of AA [31,32]. Baur et al. (2015) improved the adsorption performance of AA by supporting metal oxides such as La_2_O_3_, CaO, MgO, ZnO, Al_2_O_3_, and TiO_2_ on the surface of ACF, and MgO and ZnO increased the AA adsorption capacity by about 4 to 5 times [31]. In this study, the Ag/ACF composites prepared by the LPP method also showed an increase in the amount of AA adsorption at the breakthrough time (tb), similar to the results of the above study.

The breakthrough curve showing the dynamic adsorption characteristics between adsorbent–adsorbate systems is used as important information to design an actual fixed bed adsorption column [33]. In this study, Bohart–Adams, Thomas, Yoon–Nelson, Dose–Response, and Clark equations were used to examine the dynamic adsorption characteristics of AA in a fixed bed column [34,35,36,37,38]. Although the above-mentioned equations are mathematically simple and have limited model parameters, they are widely used in fixed-bed column breakthrough curve modeling studies because they show excellent fitting results [33,39]. The parameter values of each of the above-mentioned formulas were obtained using the Nelder–Mead method [40]. Table 4 shows the parameter values and correlation coefficient (R^2^) values of each equation. Among the formulas used, the Dose–Response model showed the highest correlation coefficient (R^2^ > 0.994) compared to other models, and the fitting for the results in Figure 5 was in the order Dose–Response > Clark > Bohart–Adams = Thomas = Yoon–Nelson. Through the above results, it can be said that the AA adsorption of ACF and Ag/ACF composites is suitable for the Dose–Response model. It is known that the Dose–Response model describes a system in which diffusion within particles is a rate controlling step, and the adsorbent is composed of two or more components with different reactivity, resulting in an asymmetric breakthrough curve shape [33].

**Figure 9:**
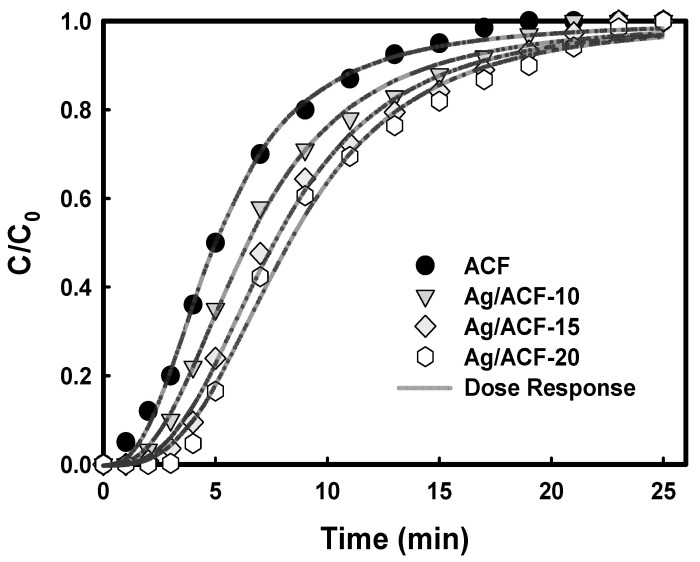
The experimental value and predicted breakthrough curve for AA adsorption based on dose response model.

Figure 9 is a graph fitted by applying the Dose–Response model to the results of Figure 7, and satisfactorily fitting the experimental data showing the asymmetric shape of the AA adsorption breakthrough curve for ACF and Ag/ACF composites.

## 4. Conclusions

Ag particles were precipitated on an ACF surface using the LPP method to prepare a Ag/ACF composite. In addition, the prepared Ag/ACF composite was applied as an adsorbent, and the efficiency was examined through an adsorption experiment of acetaldehyde. FE-SEM and EDS confirmed that Ag particles were distributed uniformly over the ACF surface. XRD showed that metallic silver (Ag^0^) and silver oxide (Ag_2_O) precipitated simultaneously on the ACF surface. High-resolution XPS confirmed peaks due to Ag_2_O and Ag^0^. The surface area of the Ag/ACF composite measured by BET was 1316 m^2^/g, which was lower than that of ACF (1462 m^2^/g). Hence, the presence of Ag reduced the surface area by blocking the pores with the Ag particles. Ag precipitated on the surface of ACF decreased the surface area and pore volume but improved the adsorption properties of acetaldehyde. The adsorption affinity between the Ag particles precipitated on the surface of the ACF and the carbonyl group of AA is increased, and it is presumed that the adsorption capacity is improved. The AA adsorption of ACF and Ag/ACF composites performed in this study was suitable for the Dose–Response model, and the experimental data showing the asymmetric shape of the AA adsorption breakthrough curve for ACF and Ag/ACF composites were satisfactorily fitted.

## Figures and Tables

**Table 1:** Atomic composition of ACF prepared from high-density polyethylene fiber and Ag/ACF composites prepared by LPP method.

Sample	Initial Conc. of Precursor (mM)	Atomic Composition (%)		
		C	O	Ag
ACF	0	96.57	3.43	0.00
Ag/ACF-10	10	93.63	4.84	1.53
Ag/ACF-15	15	92.11	5.42	2.47
Ag/ACF-20	20	90.92	5.81	3.27

**Table 2:** Textural properties of ACF and Ag/ACF composites.

Sample	S_BET_ ^1^ (m^2^/g)	V_Total_ ^2^ (cm^3^/g)	V_Micro_ ^3^ (cm^3^/g)	V_Meso_ ^4^ (cm^3^/g)	Mesopore Ratio ^5^ (%)	Pore Diameter ^6^ (nm)
ACF	1462	0.6844	0.4856	0.1988	29.05	4.42
Ag/ACF-10	1316	0.6438	0.4771	0.1667	25.89	3.96
Ag/ACF-15	1264	0.6296	0.4734	0.1562	24.80	4.03
Ag/ACF-20	1208	0.6127	0.4702	0.1425	23.52	4.02

^1^ S_BET_: Specific surface area; BET method, ^2^ V_Total_: Total pore volume; P/P_o_ = 0.990. ^3^ V_Micro_: Micropore volume; t-plot method, ^4^ V_Meso_: Mesopore volume; V_Tota_l − V_Meso_. ^5^ Mesorpore ratio: (V_meso_/V_Total_) × 100. ^6^ Pore diameter: BJH method.

**Table 3:** Comparison of efficiency parameters for acetaldehyde adsorption.

Sample	t_b_ ^1^ (min)	t_e_ ^2^ (min)	Q_b_ ^3^ (mg/g)	Q_e_ ^4^ (mg/g)	%R_b_ ^5^	%R_e_ ^6^	h__MTZ_ ^7^ (cm)
ACF	1.0	19.0	26.0	161.6	97.5	31.9	0.42
Ag/ACF-10	2.4	21.0	63.1	207.5	98.6	37.1	0.35
Ag/ACF-15	3.5	23.0	92.1	238.8	98.7	38.9	0.31
Ag/ACF-20	4.1	25.0	108.6	258.7	99.4	38.8	0.29

^1^ Breakthrough time, ^2^ Exhaustion time. ^3^ Breakthrough adsorption capacity: $Q_{b}=\frac{C_{0}Q}{m}\int_{0}^{t_{b}}\left(1-\frac{C}{C_{0}}\right)dt$. ^4^ Exhaustion adsorption capacity: $Q_{e}=\frac{C_{0}Q}{m}\int_{0}^{\infty }\left(1-\frac{C}{C_{0}}\right)dt$, where m is the adsorbent mass (g) and t is the operating time. ^5^ Removal percentages of breakthrough: $\%R_{b}=\left(\frac{q_{b}m}{C_{0}Qt_{b}}\right)\times 100$. ^6^ Removal percentages of exhaustion:$\;\%R_{e}=\left(\frac{q_{e}m}{C_{0}Qt_{e}}\right)\times 100$. ^7^ Height of the mass transfer zone: $h_{\mathrm{MTZ}}=\left(1-\frac{q_{b}}{q_{e}}\right)Z$.

**Table 4:** Breakthrough models used in this study and their calculated parameters.

Model	Parameter	ACF	Ag/ACF-10	Ag/ACF-15	Ag/ACF-20
Bohart–Adams ^1^	k_BA_ (L/mg min)	2.443 × 10^−2^	2.044×10^−2^	1.936 × 10^−2^	1.876 × 10^−2^
	N_0_ (mg/L)	1.018 × 10^5^	1.101×10^5^	1.216 × 10^5^	1.272 × 10^5^
	R^2^	0.989	0.979	0.977	0.972
Thomas ^2^	k_Th_ (L/mg min)	2.443 × 10^−2^	2.044 × 10^−2^	1.936 × 10^−2^	1.876 × 10^−2^
	Q_0_ (mg/g)	146.9	158.9	175.5	183.6
	R^2^	0.989	0.979	0.977	0.972
Yoon–Nelson ^3^	k_YN_ (1/min)	5.509	7.123	8.308	8.968
	τ (min)	0.489	0.409	0.387	0.375
	R^2^	0.989	0.979	0.977	0.972
Dose–Response ^4^	a	2.565	2.652	2.902	3.001
	Q_F_(mg/g)	133.3	171.6	203.0	220.9
	R^2^	0.997	0.997	0.996	0.994
Clark ^5^	B	0.288	0.247	0.163	0.115
	r (1/min)	0.339	0.278	0.263	0.251
	n	1.067	1.052	1.029	1.019
	R^2^	0.997	0.992	0.991	0.989

^1^ Bohart–Adams: $\frac{C}{C_{0}}=\frac{1}{1+\exp\left(\frac{k_{BA}N_{0}L}{u}-k_{BA}C_{0}t\right)}$, ^2^ Thomas: $\frac{C}{C_{0}}=\frac{1}{1+\exp\left(\frac{k_{Th}q_{0}m}{Q}-k_{Th}C_{0}t\right)}.\;$^3^ Yoon–Nelson: $\frac{C}{C_{0}}=\frac{1}{1+\exp\left(k_{YN}\tau -k_{YN}t\right)}$, ^4^ Dose–Response:$\;\frac{C}{C_{0}}=1-\frac{1}{1+{\left(Q_{F}t\right)}^{a}}$. ^5^ Clark: $\frac{C}{C_{0}}={\left(\frac{1}{1+B\exp\left(-rt\right)}\right)}^{\frac{1}{n-1}}$.

## Data Availability

The data presented in this study are available on request from the corresponding author.
